# Different Physical Activity Subtypes and Risk of Metabolic Syndrome in Middle-Aged and Older Chinese People

**DOI:** 10.1371/journal.pone.0053258

**Published:** 2013-01-07

**Authors:** Mu Chen, Meian He, Xinwen Min, An Pan, Xiaomin Zhang, Ping Yao, Xiulou Li, Yuewei Liu, Jing Yuan, Weihong Chen, Li Zhou, Weimin Fang, Yuan Liang, Youjie Wang, Xiaoping Miao, Mingjian Lang, Peng Zhang, Dongfeng Li, Huan Guo, Handong Yang, Frank B. Hu, Tangchun Wu

**Affiliations:** 1 MOE Key Lab of Environment and Health, School of Public Health, Tongji Medical College, Huazhong University of Science and Technology, Wuhan, Hubei, China; 2 Departments of Nutrition and Epidemiology, Harvard School of Public Health, Boston, Massachusetts, United States of America; 3 Dongfeng Central Hospital, Dongfeng Motor Corporation and Hubei University of Medicine, Shiyan, Hubei, China; The University of Texas M. D. Anderson Cancer Center, United States of America

## Abstract

**Background:**

The prevalence of metabolic syndrome (MetS) is growing rapidly in China. Tai chi and dancing are common types of exercise among middle-aged and elderly Chinese. It remains unclear whether these activities are associated with a lower risk of MetS.

**Methodology/Principal Findings:**

A total of 15,514 individuals (6,952 men, 8,562 women) aged 50 to 70 years from the Dongfeng-Tongji Cohort in Shiyan, China participated in a cross-sectional study. Physical activity and other lifestyle factors were assessed with semi-structured questionnaires during face-to-face interviews. MetS was defined by the current National Cholesterol Education Program/Adult treatment Panel III criteria for Asian Americans. The prevalence of MetS was 33.2% in the study population. In the multivariable-adjusted logistic regression analyses, total physical activity levels were monotonically associated with a lower odds of MetS [OR 0.75 comparing extreme quintiles, 95% confidence interval (CI) 0.66–0.86, *P*<0.001]. Compared with non-exercisers in a specific exercise type, jogging (OR 0.82, 95% CI 0.68–1.00, *P* = 0.046), tai chi (OR 0.72, 95% CI 0.60–0.88, *P*<0.001), and dancing (OR 0.56, 95% CI 0.47–0.67, *P*<0.001) were associated with significantly lower odds of MetS. Furthermore, each 1–h/week increment in tai chi and dancing was associated with a 5% (95% CI 2%–9%) and a 9% (95% CI 6%, 12%) lower risk of MetS.

**Conclusions/Significance:**

Jogging, tai chi and dancing are associated with a significantly lower risk of having MetS in middle-aged and older Chinese. Future intervention studies should consider the role of jogging, tai chi and dancing in preventing MetS.

## Introduction

With rapid industrialization and urbanization, China is undergoing an epidemiologic transition of disease burdens with a substantial increase in cardiovascular diseases (CVD), which are now the leading cause of death in China [1]. Metabolic syndrome (MetS) is defined as a cluster of risk factors (central obesity, hypertension, dyslipidemia, and hyperglycemia) [2] and plays a critical role in the development of type 2 diabetes and CVD [3], [4]. Studies have consistently shown an inverse association between physical activity levels and risk of MetS [5], [6], [7], and physical inactivity has been indicated as one of the most important preventable risk factors in China [8]. However, most previous research has focused on overall physical activity levels and specific physical activity types were not assessed, and thus the relations of physical activity subtypes with MetS remain unclear.

Certain specific types of exercise, such as tai chi and dancing, are popular among middle-aged and older Chinese people. Tai chi, the Chinese martial art involving slow and rhythmic movement, has been shown to help people maintain balance and strength [9], [10]. Recent studies found that tai chi might be a useful treatment for fibromyalgia [11] and Parkinson’s disease [12] most likely due to its effects on enhancing physical, psychological, and psychosocial well-being and overall quality of life. A randomized controlled trial also suggested that tai chi improved body mass index (BMI), serum lipid profile, C-reactive protein (CRP), and oxidative stress levels [13]. Dancing is a unique phenomenon in China and middle-aged and older people gather together to dance in open areas such as parks and streets. Aerobic dancing has been found to be significantly associated with lower levels of inflammatory markers such as CRP and fibrinogen [14]. However, it remains unclear whether tai chi and dancing are associated with MetS.

Since tai chi and dancing are unique and relatively common types of exercise among middle-aged and older Chinese, the aim of this study was to examine the associations of different physical activity subtypes with risk of MetS in a middle-aged and older Chinese population.

## Materials and Methods

### Study Participants

The Dongfeng-Tongji Cohort (DFTJ cohort) study was launched in 2008 among retirees of Dongfeng Motor Corporation (DMC) in Shiyan City, Hubei province [15]. DMC was founded in 1969 and is one of the 3 largest auto manufacturers in China. Between 2008 and 2010, 87% (n = 27,009 out of 31,000) of retired employees who agreed to answer the questionnaires and provide baseline blood samples were recruited. For this study, participants were excluded if they were younger than 50 years old, had a history of cancer, diabetes, CVD, or stroke, had missing data on one of the exercise subtypes, on one component of MetS diagnostic, or one of the demographic variables (age, sex, and education).

### Data Collection

Baseline data were collected by trained interviewers via semi-structured questionnaires during face-to-face interviews. The questionnaire was designed based on 6 pilot surveys among this population. Information on socio-demographic factors, health status, and lifestyle practices (including dietary factors and physical activity) was included in the questionnaire. Standing height, body weight, and waist circumference were measured with participants in light indoor clothing and without shoes. Body mass index was calculated as weight in kilograms divided by height in meters squared. Two researchers independently entered the baseline data from questionnaires and data were further checked by a third researcher when differences were found.

All subjects were examined in the morning after an overnight fast. Fasting blood was drawn with a vacuum coagulation tube for serum, with five milliliters in the tube. Serum triglyceride, blood glucose, and high-density lipoprotein (HDL) cholesterol were measured by the hospital’s laboratory using ARCHITECT ci8200, Abbott, USA.

### Assessment of Physical Activity and Covariates

Frequency and average duration of each type of physical activity were obtained via the questionnaires: walking, biking, tai chi, jogging, swimming, dancing, climbing stairs, playing basketball, volleyball, or soccer. To estimate energy expenditure for total physical activity, separate metabolic equivalent (MET) hours per week were calculated for each activity according to the following formulas: MET coefficient of activity×duration (hours per time) × frequency (times per week). Using the compendium of physical activities, METs per hour used for leisure activities were: 3 for walking, 4 for biking, 4.5 for tai chi [16], 7.5 for jogging or swimming, 5 for dancing, 4.5 for climbing, 6 for playing ball games or doing exercise in gym [17]. Physical activity levels were categorized into five groups according to quintiles of gender-specific distribution: ≤9.0, 9.1–21.0, 21.1–28.5, 28.6–45.0, and >45.0 MET-h/wk for men; ≤6.8, 6.9–18.0, 18.1–24.5, 24.6–42.0, and >42.0 MET-h/wk for women.

Educational attainment was categorized as low (0 to 6 years), medium (7 to 9 years), and high (≥10 years). According to the respondents’ self-reported smoking status, participants were grouped as current smokers, ex-smokers, and nonsmokers. Other variables were dichotomized as yes or no on the basis of the responses to questions on current use of alcohol, antihypertensive drug, aspirin, and antibiotics, and family history of CVD, diabetes mellitus and stroke.

### Definition of the Metabolic Syndrome

MetS was defined using the updated National Cholesterol Education Program/Adult treatment Panel III criteria for Asian Americans as having ≥3 of the following components: waist circumference ≥90 cm for men or ≥80 cm for women; triglycerides (TG) ≥1.7 mmol/L; high density lipoprotein (HDL) cholesterol <1.03 mmol/L for men or <1.30 mmol/L for women; blood pressure ≥130/85 mmHg or current use of antihypertensive medications; or fasting glucose ≥5.6 mmol/L [18].

### Ethical Considerations

The study was approved by the Medical Ethics Committee of the School of Public Health, Tongji Medical College, and Dongfeng General Hospital, DMC. All participants provided written informed consent.

### Statistical Analysis

All statistical analyses were performed using SAS 9.2 software (SAS institute, Cary, NC). Categorical variables were expressed in percentages and continuous variables were expressed in means ± SD for normally distributed data or medians (interquartile ranges) for skewed parameters. Differences in the variables by different levels and types of physical activity were assessed by either a general linear regression model for continuous variables or a logistic regression model for categorical variables. Multivariable adjusted odds ratios (ORs) and 95% confidence intervals (CIs) for MetS and its components with the logistic regression model were computed. The multivariable model adjusted for age, sex, education, smoking, alcohol drinking, use of antihypertensive drugs, aspirin use, the use of glucose lowering or lipid-lowering medications, family history of CVD, stroke, and diabetes mellitus, and several dietary factors (daily intakes of meat, fruit, vegetable, and nuts, all in quintiles). A two-sided *P* value <0.05 was considered significant.

## Results

### Descriptive Characteristics

Based on exclusion criteria, 719 participants younger than 50 years old, 1463 cancer patients, 3197 diabetics, 4343 CVD patients, 1128 stroke patients, 3698 participants with missing data on one of the exercise subtypes or one component of MetS diagnostic criteria, and 390 participants with one of the demographic variables missing were excluded. After excluding these participants, a total of 15,514 individuals (6952 men, 8562 women) were included in this study. The prevalence of MetS was 33.2% in the total sample, 27.4% in men, and 37.8% in women. Characteristics of the study population according to quintiles of physical activity levels are summarized in Table 1. Individuals with higher levels of total physical activity were less likely to be current smokers, and more likely to be current drinkers, use aspirin, and have a lower body mass index and waist circumference.

**Table 1:** Characteristics of the study participants according to total physical activity quintiles.

	Quintile of MET					
	Quintile 1(n = 3039)	Quintile 2(n = 2966)	Quintile 3(n = 3315)	Quintile 4(n = 2998)	Quintile 5(n = 3196)	*P*_for trend_
**Age (years)**	62.7±7.4	63.3±7.5	63.6±7.3	63.3±7.3	62.9±6.9	0.48
**Female, n (%)**	1715 (56.4)	1647 (55.5)	1787 (53.9)	1379 (46.0)	1792 (56.1)	0.49
**Body mass index (kg/m^2^)**	24.4 (21.9–26.7)	24.3 (21.9–26.4)	24.5 (22.2–26.7)	24.2 (22.1–26.3)	24.2 (22.0–26.2)	0.002
**Education, n (%)**						0.08
0–6 y	940 (30.9)	783 (26.4)	1029 (31.0)	869 (29.0)	983 (30.8)	
7–9 y	1107 (36.5)	1056 (35.6)	1228 (37.0)	1101 (36.7)	1200 (37.6)	
≥10 y	992 (32.6)	1127 (38.0)	1058 (31.9)	1028 (34.3)	1013 (31.7)	
**Smoking status, n (%)**						0.02
Nonsmokers	2109 (70.0)	2106 (71.5)	2302 (70.1)	2122 (71.2)	2267 (71.4)	
Ex-smokers	254 (8.4)	293 (10.0)	349 (10.6)	311 (10.4)	347 (10.9)	
Current smokers	649 (21.6)	546 (18.5)	634 (19.3)	548 (18.4)	562 (17.7)	
**Alcohol drinking, n (%)**						0.004
Not current drinkers	2340 (77.2)	2314 (78.0)	2577 (77.7)	2335 (77.9)	2372 (74.3)	
Current drinkers	691 (22.8)	651 (22.0)	737 (22.2)	661 (22.1)	822 (25.7)	
**Family history, n (%)**						
CVD	151 (5.0)	140 (4.7)	130 (3.9)	123 (4.1)	154 (4.8)	0.47
Stroke	116 (3.8)	156 (5.3)	128 (3.9)	147 (4.9)	148 (4.6)	0.28
Diabetes	95 (3.1)	125 (4.2)	106 (3.1)	123 (4.1)	132 (4.1)	0.07
**Waist (cm)**	83.0 (76.0–90.0)	82.4 (76.0–89.0)	82.7 (76.0–89.0)	82.0 (76.0–88.0)	81.9 (75.0–88.0)	0.001
**TG (mmol/L)**	1.43 (0.86–1.68)	1.40 (0.87–1.68)	1.44 (0.86–1.73)	1.38 (0.84–1.60)	1.34 (0.80–1.61)	<0.001
**HDL (mmol/L)**	1.46 (1.19–1.66)	1.46 (1.20–1.66)	1.45 (1.19–1.65)	1.44 (1.19–1.64)	1.47 (1.20–1.68)	0.61
**SBP (mmHg)**	128.3 (115.0–140.0)	128.1 (115.0–150.0)	129.4 (120.0–140.0)	128.4 (120.0–140.0)	128.4 (120.0–140.0)	0.62
**DBP (mmHg)**	78.0 (70.0–85.0)	77.3 (70.0–80.0)	77.9 (70.0–85.0)	77.4 (70.0–80.0)	77.6 (70.0–80.0)	0.35
**Glucose (mmol/L)**	5.8 (5.2–6.0)	5.8 (5.2–6.1)	5.8 (5.2–6.0)	5.8 (5.2–6.0)	5.7 (5.2–6.0)	0.42
**Glucose lowering drug use, n (%)**	23 (0.8)	22 (0.7)	29 (0.9)	34 (1.1)	33 (1.0)	0.08
**Lipid–lowering drug use, n (%)**	196 (6.5)	196 (6.5)	256 (7.7)	196 (6.5)	217 (6.8)	0.76
**Antihypertension drug use, n (%)**	647 (21.3)	691 (23.3)	825 (24.9)	725 (24.2)	765 (23.9)	0.012
**Aspirin drug use, n (%)**	204 (6.7)	266 (9.0)	288 (8.7)	269 (9.0)	286 (9.0)	0.005
**MET (hours/week)**	1.7 (0–3.5)	12.6 (10.5–15.0)	22.0 (21.0–23.0)	33.8 (30.0–38.0)	65.4 (49.0–77.0)	<0.001

Data are mean ± SD for normally distributed or medians (interquartile ranges) for skewed parameters, or number (%).

### Relations between Physical Activity Subtype and the MetS

**Figure 1:**
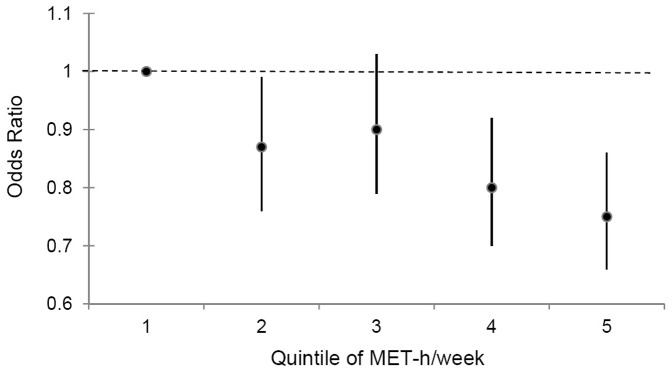
Odd ratios (95% CIs) of MetS associated with quintile of MET-h/week. Odds ratios were estimated by multivariate logistic regression adjusted for age, sex, education levels, smoking, alcohol drinking, use of antihypertension drugs, aspirin, glucose lowering drugs and lipid-lowering drugs, family history of CVD, stroke and diabetes mellitus, and meat intake quintile, fruit and vegetable intake quintile, and consumption of nuts quintiles. Solid round spots denote the point estimates of the odds ratio and vertical lines represent 95% CIs.

Multivariate logistic regression model indicated that total physical activity levels were inversely associated with risk of MetS (Q5 vs. Q1 OR 0.75, 95% CI 0.66–0.86; *P* for trend <0.001), as displayed in Figure 1. The association between specific types of physical activity and risk of MetS (Table 2) was further assessed. In this analysis, all the subtypes of physical activity were entered into the model simultaneously. Brisk walking was the most common type of physical activity (80.3% of the participants reported that they walked regularly, n = 12437). The percentage of regularly participating in other types of physical activity ranged from 0.9% for swimming to 18.8% (n = 2923) for climbing mountains or stairs. Approximately 6.2% (n = 962) and 8.0% (n = 1236) of participants engaged in tai chi and dancing, respectively. Among those who reported specific types of activity, the mean weekly duration of exercise subtypes varied from 3.1 hrs/week for swimming to 6.0 hrs/week for walking, with most weekly durations being around 4 hrs/week. In the multivariable-adjusted model, only jogging, tai chi and dancing were found to be significantly associated with lower odds of MetS, and the respective corresponding ORs were 0.82 (95% CI 0.68–1.00), 0.72 (95% CI 0.60–0.88) and 0.56 (95% CI 0.47–0.67), comparing people who performed jogging, tai chi and dance with participants who did not.

**Table 2:** Odds ratio and 95% CI for MetS by types of physical activity.

Subtype ofphysical activity	No (%)	Frequency(hrs/wk)	MetS, Model 1^a^		MetS, Model 2^b^	
			OR (95% CI)	*P*	OR (95% CI)	*P*
Walking	12437 (80.2)	6.0±4.9	1.03 (0.95, 1.12)	0.51	0.90 (0.80, 1.03)	0.12
Biking	1160 (7.5)	3.9±3.3	0.89 (0.77, 1.02)	0.084	0.91 (0.76, 1.09)	0.29
Dancing	1236 (8.0)	4.5±3.4	0.59 (0.52, 0.68)	<0.001	0.56 (0.47, 0.67)	<0.001
Tai chi	962 (6.2)	4.0±3.0	0.71 (0.61, 0.82)	<0.001	0.72 (0.60, 0.88)	0.001
Gym	492 (3.2)	3.3±2.4	0.96 (0.76, 1.12)	0.65	1.00 (0.78, 1.28)	0.99
Ball games	959 (6.2)	4.2±3.3	0.81 (0.70, 0.94)	0.006	0.91 (0.75, 1.09)	0.30
Jogging	948 (6.1)	3.9±3.0	0.79 (0.68, 0.92)	0.002	0.82 (0.68, 1.00)	0.046
Swimming	145 (0.9)	3.1±2.4	0.88 (0.60, 1.28)	0.50	0.82 (0.50, 1.34)	0.42
Climbing	2923 (18.8)	4.3±3.4	0.99 (0.90, 1.08)	0.76	1.06 (0.95, 1.19)	0.29

a Model 1: adjusted for age, sex, education levels. Physical activity subtypes were treated as bivariate and nonexercisers in the subtype were set as reference group.

b Model 2: additionally adjusted for smoking, alcohol drinking, use of antihypertension drugs, aspirin, glucose lowering drugs and lipid-lowering drugs, family history of CVD, stroke and diabetes mellitus, and meat intake quintile, fruit and vegetable intake quintile, and consumption of nuts quintile.

The association between weekly duration of activity subtypes and risk of MetS was also examined. In the multivariate logistic regression, each 1-h/week increment in tai chi and dancing was associated with a 5% (95% CI 2%–9%) and a 9% (95% CI 6%–12%) lower risk of MetS; while the other subtypes were not statistically significant. Moreover, by categorizing exercise frequencies into 5 groups (0, 0.1–2.0, 2.1–3.5, 3.6–6.0, >6.0 hrs/week), only dancing (*P* for trend<0.001), tai chi (*P* for trend = 0.002), and jogging (*P* for trend = 0.044) showed a significant inverse dose-response relationship with risk of having MetS (Table 3).

**Table 3:** Odds ratio and 95% CI for MetS according to categories of physical activity subtypes.

Physical activity subtypes	Physical activity levels (hrs/wk)					*P* for Trend	Continuous
	0	0.1–2.0	2.1–3.5	3.6–6.0	>6.0		
Dancing							
No. of cases	4845	77	92	75	55		
No. of persons	14310	275	342	320	267		
Model 1^a^ OR (95% CI)	1.00	0.72 (0.55, 0.95)	0.65 (0.51, 0.83)	0.55 (0.42, 0.72)	0.46 (0.34, 0.62)	<0.001	0.92 (0.89, 0.94)
Model 2^b^ OR (95% CI)	1.00	0.59 (0.40, 0.86)	0.68 (0.50, 0.93)	0.54 (0.39, 0.75)	0.45 (0.31, 0.65)	<0.001	0.91 (0.88, 0.94)
Tai chi							
No. of cases	4877	61	104	48	54		
No. of persons	14570	227	338	197	182		
Model 1^a^ OR (95% CI)	1.00	0.66 (0.49, 0.89)	0.80 (0.63, 1.01)	0.59 (0.42, 0.82)	0.74 (0.54, 1.03)	<0.001	0.95 (0.92, 0.98)
Model 2^b^ OR (95% CI)	1.00	0.59 (0.40, 0.88)	0.83 (0.61, 1.13)	0.62 (0.41, 0.94)	0.71 (0.47, 1.10)	0.002	0.95 (0.91, 0.98)
Biking							
No. of cases	4846	88	88	50	54		
No. of persons	14397	328	348	226	215		
Model 1^a^ OR (95% CI)	1.00	0.90 (0.70, 1.16)	1.08 (0.85, 1.36)	0.70 (0.51, 0.96)	0.86 (0.63, 1.17)	0.089	0.97 (0.95, 1.00)
Model 2^b^ OR (95% CI)	1.00	0.88 (0.63, 1.22)	1.23 (0.91, 1.65)	0.78 (0.52, 1.18)	0.72 (0.47, 1.11)	0.236	0.97 (0.93, 1.01)
Walking							
No. of cases	1080	619	937	738	1770		
No. of persons	3347	1963	2895	2277	5032		
Model 1^a^ OR (95% CI)	1.00	0.97 (0.86, 1.10)	0.98 (0.88, 1.09)	1.00 (0.89, 1.12)	1.08 (0.98, 1.19)	0.061	1.01 (1.00, 1.02)
Model 2^b^ OR (95% CI)	1.00	0.86 (0.73, 1.02)	0.90 (0.76, 1.06)	0.93 (0.77, 1.11)	0.94 (0.78, 1.14)	0.456	1.01 (1.00, 1.02)
Gym							
No. of cases	4997	55	52	22	18		
No. of persons	15034	160	166	92	62		
Model 1^a^ OR (95% CI)	1.00	1.11 (0.79, 1.54)	0.95 (0.68, 1.32)	0.69 (0.43, 1.12)	0.89 (0.51, 1.55)	0.261	0.97 (0.92, 1.02)
Model 2^b^ OR (95% CI)	1.00	1.32 (0.88, 1.97)	0.99 (0.65, 1.50)	0.52 (0.27, 0.99)	0.98 (0.49, 2.00)	0.418	0.98 (0.92, 1.04)
Ball games							
No. of cases	4881	70	79	56	58		
No. of persons	14569	268	271	202	204		
Model 1^a^ OR (95% CI)	1.00	0.79 (0.60, 1.04)	0.83 (0.63, 1.08)	0.80 (0.59, 1.10)	0.82 (0.60, 1.11)	0.013	0.97 (0.94, 1.00)
Model 2^b^ OR (95% CI)	1.00	0.95 (0.66, 1.35)	0.92 (0.65, 1.30)	0.95 (0.64, 1.42)	0.78 (0.53, 1.15)	0.207	0.97 (0.94, 1.01)
Jogging							
No. of cases	4903	73	75	46	47		
No. of persons	14588	266	294	187	179		
Model 1^a^ OR (95% CI)	1.00	0.86 (0.66, 1.13)	0.75 (0.58, 0.99)	0.73 (0.52, 1.02)	0.80 (0.57, 1.12)	0.003	0.97 (0.94, 0.97)
Model 2^b^ OR (95% CI)	1.00	0.90 (0.64, 1.26)	0.80 (0.57, 1.13)	0.77 (0.50, 1.19)	0.78 (0.50, 1.21)	0.044	0.97 (0.93, 1.01)
Swimming							
No. of cases	5106	18	11	7	2		
No. of persons	15369	60	42	25	18		
Model 1^a^ OR (95% CI)	1.00	1.06 (0.61, 1.86)	0.88 (0.44, 1.77)	1.04 (0.43, 2.50)	0.27 (0.06, 1.20)	0.226	0.92 (0.83, 1.02)
Model 2^b^ OR (95% CI)	1.00	1.14 (0.56, 2.35)	1.38 (0.57, 3.34)	0.39 (0.12, 1.32)	0.17 (0.02, 1.35)	0.095	0.88 (0.77, 1.01)
Climbing							
No. of cases	4254	216	289	206	179		
No. of persons	12658	698	925	630	603		
Model 1^a^ OR (95% CI)	1.00	1.00 (0.85, 1.18)	0.98 (0.85, 1.14)	1.07 (0.90, 1.27)	0.91 (0.76, 1.09)	0.657	0.99 (0.98, 1.01)
Model 2^b^ OR (95% CI)	1.00	1.10 (0.89, 1.37)	1.06 (0.88, 1.28)	1.13 (0.90, 1.41)	0.93 (0.72, 1.19)	0.570	0.99 (0.97, 1.02)

a Model 1: adjusted for age, sex, education levels.

b Model 2: additionally adjusted for smoking, alcohol drinking, use of antihypertension drugs, aspirin, glucose lowering drugs and lipid-lowering drugs, family history of CVD, stroke and diabetes mellitus, and meat intake quintile, fruit and vegetable intake quintile, consumption of nuts quintile, and other physical activity types.

Table 4 showed the associations of weekly exercise duration with biomarkers, blood pressures, and waist circumference. Total physical activity was associated with lower TG and blood glucose levels; each 5 MET-hrs/week increase was associated with 0.009 mmol/L (95% CI 0.005–0.013) decrease in TG, 0.008 mmol/L (95% CI 0.003–0.013) decrease in blood glucose, and 0.1 cm decrease in waist circumference. Furthermore, each 1-h/week increment in dancing was significantly associated with lower TG (−0.023 mmol/L, 95% CI −0.035–0.012, *P*<0.001), blood glucose (−0.024 mmol/L, 95% CI −0.040–0.008, *P* = 0.003), systolic (−0.430 mmHg, 95% CI −0.646–0.214, *P*<0.001) and diastolic blood pressures (−0.177 mmHg, 95% CI −0.308–0.046, *P* = 0.008), waist circumference (−0.305 cm, 95% CI −0.417–0.194, *P*<0.001), and higher HDL (0.008 mmol/L, 95% CI 0.003–0.013, *P* = 0.003). For tai chi, each 1-h/week increment was associated with lower TG (−0.023 mmol/L, 95% CI −0.039–0.008, *P* = 0.003), blood glucose (−0.014 mmol/L, 95% CI −0.034–0.006, *P = *0.17), systolic (−0.059 mmHg, 95% CI −0.341–0.223, *P = *0.68) and diastolic blood pressures (−0.198 mmHg, 95% CI −0.369–0.028, *P = *0.023), waist circumference (−0.325 cm, 95% CI −0.471–0.180, P<0.001), and higher HDL (0.004 mmol/L, 95% CI −0.003–0.011, *P = *0.22). In addition, each 1-h/week increment in jogging was significantly associated with lower TG (−0.019 mmol/L, 95% CI −0.034–0.004, *P = *0.015), lower waist circumference (−0.219 mmol/L, 95% CI −0.362–0.075, *P = *0.003), and higher HDL (0.008 mmol/L, 95% CI 0.001–0.0014, *P = *0.023).

**Table 4:** Differences in biomarkers and blood pressure by physical activity subtypes.

	TG (mmol/L)	HDL (mmol/L)	Glucose (mmol/L)	SBP (mm Hg)	DBP (mm Hg)	WC (cm)
MET^a^	−0.009(−0.013, −0.005)	0.001(−0.001, 0.003)	−0.008(−0.013, −0.003)	−0.021(−0.090, 0.049)	−0.038(−0.080, 0.004)	−0.099(−0.135, −0.063)
* P*	<0.0001	0.15	0.003	0.56	0.07	<0.001
Walking^b^	−0.001(−0.005, 0.003)	−0.001(−0.002, 0.001)	0.002(−0.003, 0.007)	0.009(−0.061, 0.079)	−0.015(−0.058, 0.027)	−0.013(−0.049, 0.023)
* P*	0.65	0.55	0.38	0.80	0.48	0.47
Biking^b^	−0.015(−0.029, 0.001)	0.002(−0.001, 0.008)	−0.018(−0.037, 0.001)	−0.068(−0.327, 0.191)	0.047(−0.109, 0.204)	−0.029(−0.162, 0.105)
* P*	0.037	0.63	0.06	0.61	0.56	0.68
Dancing^b^	−0.023(−0.035, −0.012)	0.008(0.003, 0.013)	−0.024(−0.040, −0.008)	−0.430(−0.646, −0.214)	−0.177(−0.308, −0.046)	−0.305(−0.417, −0.194)
* P*	<0.001	0.003	0.003	<0.001	0.008	<0.001
Tai chi^b^	−0.023(−0.039, −0.008)	0.004(−0.003, 0.011)	−0.014(−0.034, 0.006)	−0.059(−0.341, 0.223)	−0.198(−0.369, −0.028)	−0.325(−0.471, −0.180)
* P*	0.003	0.22	0.17	0.68	0.023	<0.001
Gym^b^	−0.010(−0.035, 0.015)	0.013(0.002, 0.024)	−0.017(−0.034, 0.001)	0.088(−0.384, 0.559)	−0.057(−0.342, 0.227)	−0.209(−0.449, −0.032)
* P*	0.44	0.019	0.07	0.72	0.69	0.09
Ball games^b^	−0.007(−0.020, 0.006)	0.003(−0.002, 0.009)	−0.019(−0.036, −0.002)	−0.156(−0.399, 0.086)	−0.087(−0.234, 0.059)	−0.148(−0.273, −0.023)
* P*	0.30	0.24	0.030	0.21	0.24	0.020
Jogging^b^	−0.019(−0.034, −0.004)	0.008(0.001, 0.014)	−0.007(−0.027, 0.014)	−0.066(−0.344, 0.213)	−0.011(−0.179, 0.158)	−0.219(−0.362, −0.075)
* P*	0.015	0.023	0.52	0.64	0.90	0.003
Swimming^b^	−0.003(−0.049, 0.042)	0.002(−0.017, 0.022)	−0.048(−0.109, 0.014)	0.151(−0.691, 0.992)	0.167(−0.342, 0.675)	−0.193(−0.627, 0.241)
* P*	0.89	0.83	0.13	0.73	0.52	0.38
Climbing^b^	−0.006(−0.015, 0.002)	−0.004(−0.008, −0.001)	−0.017(−0.028, −0.005)	0.149(−0.006, 0.303)	0.014(−0.079, 0.107)	−0.065(−0.145, 0.014)
* P*	0.15	0.025	0.004	0.06	0.76	0.11

a For 5 MET hrs/week increase. Adjusted for age, sex, education levels, smoking, alcohol drinking, use of antihypertension drugs, aspirin, glucose lowering drugs and lipid-lowering drugs, family history of CVD, stroke and diabetes mellitus, and meat intake quintile, fruit and vegetable intake quintile, and consumption of nuts quintiles.

b For 1-h/wk increase. Adjusted for age, sex, education levels, smoking, alcohol drinking, use of antihypertension drugs, aspirin, glucose lowering drugs and lipid-lowering drugs, family history of CVD, stroke and diabetes mellitus, and meat intake quintile, fruit and vegetable intake quintile, consumption of nuts quintile, and other physical activity types.

## Discussion

We found a significant inverse association between total physical activity levels and risk of MetS among middle-aged and older Chinese people. Among subtypes of activities, the benefits of tai chi and dancing were particularly pronounced. Furthermore, there was a dose-response relationship between weekly duration (hrs/wk) of tai chi and dancing and lower risk of MetS. To the best of our knowledge, this is the first study to investigate different physical activity subtypes, especially the unique activities among middle-aged and older Chinese including tai chi and dancing, and risk of MetS.

Our results are consistent with previous studies of total physical activity levels in relation to risk of MetS [5], [6], [7]. They are also consistent with previous analyses showing benefits of moderate and vigorous intensity activity on MetS [19]. In a previous study, tai chi improved patients’ serum lipid profile including TG and HDL cholesterol [13]. Our study extended the literature showing that tai chi and dancing were associated with lower risk of MetS among middle-aged and older Chinese people. In addition, tai chi and dancing appeared to have beneficial effects on individual components of MetS including TG, HDL, blood glucose and waist circumference as well as both systolic and diastolic blood pressures.

There are several reasons that may explain the inverse associations between tai chi and dancing and risk of MetS among middle-aged and older Chinese people. First, there might be beneficial effect on psychosocial well-being from practicing tai chi and dancing. Psychological stress has been reported to be associated with higher risk of MetS [20], [21]. As a mind-body exercise, tai chi may improve immune function and reduce anxiety and mood disturbance, which can lead to enhanced physical, psychological, and psychosocial well-being and overall quality of life [22], [23]. Dancing has also been shown to produce improvements in psychological well-being and has been used as a psychotherapy [24], [25]. Furthermore, unlike individual sports like biking, jogging, and swimming, dancing is a popular group exercise among middle-aged and older Chinese; individuals form organized dancing groups and perform a variety of dances (ballroom and folk dancing are most common) in open spaces such as parks and streets in the mornings and evenings. In addition to increased physical activity levels, the group members are likely to have enhanced social support and exert positive influences on each other’s lifestyles. A recent study has shown the important effect of social networks on dietary patterns [26].

Our findings have important public health implications. The prevalence of type 2 diabetes and CVD has increased dramatically in the past several decades in countries that are undergoing rapid nutrition transitions such as China [27], [28], and the control of non-communicable diseases has become a bottleneck to China’s social and economic development [29]. It has been noted that total physical activity levels are declining dramatically in China due to urbanization [16], [30]. However, rapid industrialization and lifestyle changes may also influence the popularity of traditional Chinese exercises, such as tai chi and Chinese folk dancing. Interestingly, western ballroom dancing has increasingly gained popularity in China in recent years. Since these activities are likely to have beneficial effects on both physical health and psychosocial well-being, they can be widely recommended to the general population, especially to the middle-aged and older individuals who are at higher risk of developing MetS.

This study has several limitations. First, the cross-sectional nature of the study design limits a causal inference because it is possible that individuals who were diagnosed with chronic diseases such as hypertension or dyslipidemia might have changed their physical activity levels. However, we excluded patients with diabetes, CVD, stroke, or cancer were excluded from the analyses. Second, the role of unmeasured or residual confounding cannot be ruled out although the multivariate models adjusted for a wide range of CVD risk factors as well as several dietary factors that have been implicated in the development of MetS or its individual components. In addition, since physical activity was assessed by self-report, measurement error in the exposure variable is inevitable; but this error is likely to be non-differential and would bias the association between physical activity and the risk of developing MetS towards the null. Furthermore, caution is warranted when directly interpreting the null association for some subtypes of physical activity of which the number of participants was low, such as swimming.

Despite these limitations, this study has several notable strengths. It was the first study to examine the associations between specific subtypes of physical activity and risk of MetS in a large middle-aged and older Chinese population. Second, detailed information on unique types of physical activity such as tai chi and dancing were collected, which allowed us to examine a dose-response relationship between increasing duration of these activities and risk of MetS. Finally, we controlled a variety of relevant confounding factors in the analyses.

In conclusion, this study has shown that tai chi and dancing are associated with a significantly lower risk of MetS in a middle-aged and older Chinese population, with additional benefits as exercise frequency increases. Future intervention studies should consider the role of these activities in prevention of MetS and CVD.
